# Evidence that perceptions of and tolerance for medical ambiguity are distinct constructs: An analysis of nationally representative US data

**DOI:** 10.1111/hex.13037

**Published:** 2020-02-25

**Authors:** Nicolle Simonovic, Jennifer M. Taber, William M. P. Klein, Rebecca A. Ferrer

**Affiliations:** ^1^ Department of Psychological Sciences Kent State University Kent OH USA; ^2^ Behavioral Research Program National Cancer Institute National Institutes of Health Bethesda MD USA

**Keywords:** ambiguity aversion, conflicting information, perceived ambiguity, tolerance for ambiguity

## Abstract

**Background:**

Medical information is often conflicting and consequently perceived as ambiguous. There are individual differences both in how much people perceive ambiguity and in their tolerance for such ambiguity. Little is known about how these constructs are related to each other and with other beliefs.

**Objective:**

To examine the association between (a) perceived medical ambiguity, (b) tolerance for medical ambiguity and (c) their associations with various medical and cancer‐specific judgement and decision‐making correlates.

**Method and Participants:**

We conducted secondary data analyses using the cross‐sectional, nationally representative Health Information National Trends Survey 4, Cycle 4 (n = 3,433, 51.0% female, M_age_ = 46.5). Analyses statistically controlled for age, sex, race, education and health‐care coverage**.**

**Main variables studied:**

Perceived medical ambiguity, tolerance for medical ambiguity, cancer perceptions, health‐care experiences and preferences, and information‐seeking styles and beliefs**.**

**Results:**

Perceived medical ambiguity and tolerance for medical ambiguity were statistically independent. Higher perceived ambiguity was associated with lower perceived cancer preventability, lower reliance on doctors, lower perceived health and information‐seeking self‐efficacy, lower perceived quality of the cancer information‐seeking process, and greater cancer information avoidance. Lower tolerance for ambiguity was associated with lower cancer worry, lower trust in doctors, lower likelihood of seeking health information, and lower engagement in medical research.

**Discussion and Conclusions:**

Perceived medical ambiguity and tolerance for medical ambiguity seem to be distinct constructs. Findings have implications for how people make medical decisions when they perceive and prefer to avoid conflicting medical information.

## 1 INTRODUCTION

As scientific knowledge advances and medical technology is developed and improved, people are often faced with multiple options for medical procedures and treatments. While deciding which medical option to pursue, people might come across vague, insufficient, and contradictory information relevant to the decision, which can be perceived as ambiguous*.* Ambiguity is present when information is conflicting or there is not enough information to draw adequate conclusions. For example, mammography recommendations vary in terms of screening intervals and the age to begin screening^1^ and even personalized disease risk estimates^2^ are often imprecise (e.g., presented as a range). Ambiguity is defined as a second‐order form of uncertainty^3^ that arises from ‘a lack of reliability, credibility, or adequacy’ and may exist when the source of the information is unreliable or untrustworthy.^4^, ^5^, ^6^, ^7^

People differ in how ambiguous they perceive a particular piece of information to be. Greater perceived ambiguity has been associated with multiple avoidant and negative responses.^5^, ^6^, ^8^ Classic research on ambiguity showed that people tended to avoid choosing options perceived to have unknown probabilities.^5^, ^6^ More recent health‐related research has shown that when considering ambiguous options and information, people pessimistically rated potential risks or harms as higher and potential benefits as lower.^9^, ^10^, ^11^, ^12^ With respect to cancer, cross‐sectional data have shown that people who perceived cancer prevention information as more ambiguous (i.e., agreed with the statement, ‘There are so many different recommendations about preventing cancer, it is hard to know which ones to follow’) perceived cancer as less preventable, thought they had higher cancer risk, were more worried about cancer, and were less likely to report engaging in prevention behaviours.^11^, ^13^ Individuals enrolled in a genome sequencing trial who believed their sequencing results would be ambiguous also thought the results would be less likely to help reduce their disease risk and were less interested in learning genomic information.^9^ This general pattern of avoidance and pessimistic appraisals in response to perceived ambiguity has been labelled ‘ambiguity aversion’.

### 1.1 People differ in tolerance for versus aversion to ambiguity

In addition to research demonstrating ambiguity aversion as a general pattern of avoidance and pessimistic appraisal in response to actual and/or perceived ambiguity, researchers have examined individual differences in ambiguity aversion, or the likelihood of responding to ambiguity with avoidant and negative responses.^14^ To reduce confusion from these multiple uses of the term ‘ambiguity aversion’, we use the term ‘tolerance for ambiguity’ hereafter when referring to these individual differences. We conceptualized tolerance for ambiguity and ambiguity aversion as two endpoints on a bipolar scale. Put differently, we conceptualized lower tolerance for ambiguity as synonymous with greater ambiguity aversion.

Individual differences in tolerance for ambiguity in medical contexts have been demonstrated in focus groups^15^ and assessed quantitatively with the 6‐item Medical Ambiguity Aversion scale^14^ (AA‐Med). Few studies have used the AA‐Med scale: in one, individuals with lower tolerance for ambiguity from medical tests and treatments about which there are conflicting medical opinions reported more ambivalence about cancer screenings and perceived cancer screening as having lower benefits and greater harms.^16^ Similarly, adults in a genome sequencing trial who reported lower tolerance for medical ambiguity reported higher perceived health harms of genome sequencing and lower intentions to learn genome sequencing results.^9^ In general, these results suggest that, similar to perceiving greater ambiguity, lower tolerance for ambiguity is associated with more negative appraisals and avoidance.

### 1.2 Current study and hypotheses

The goal of this study was to examine associations between perceived medical ambiguity and tolerance for medical ambiguity, and associations of these constructs with medical and cancer‐specific judgement and decision‐making correlates. Perceived ambiguity is a judgement about the information itself (i.e., Is this information ambiguous?), whereas ambiguity aversion refers to one's reaction to the information (i.e., Can I tolerate this ambiguous information?) and is thus an attribution made by the person perceiving ambiguity. These constructs could be conceptually distinct such that people could perceive ambiguity and not be averse to it and could also be averse to ambiguity in general but not perceive it from a particular source. However, there could also be overlap among the constructs such that people who are more averse to ambiguity, and thus have lower tolerance for it, might be more sensitive to ambiguity and thus more likely to perceive it. Understanding how these constructs are related could inform interventions to promote informed decision making when medical information and recommendations are conflicting. Depending on whether and how these constructs are related, it may be important to target perceptions of the ambiguity and/or individuals’ reactions to ambiguous information.

Ambiguity can arise from multiple sources (e.g., imprecise information or lack of information^3^), and in this study, we examined perceptions of and tolerance for ambiguity from conflicting medical information. Whereas the target of the conflicting medical information differed across constructs—we measured perceived ambiguity about cancer prevention and tolerance for ambiguity about medical tests and treatments—the overarching construct was ambiguity arising from conflicting information, either from different recommendations or from experts’ conflicting opinions. We predicted that people would respond to conflicting medical information in a particular manner, regardless of the exact target (i.e., cancer prevention vs. medical tests and treatments). In a review of research on conflicting health information across a range of medical topics,^17^ researchers found that conflicting health information can have negative effects on variables relevant to health behaviour, such as intentions and risk perceptions. Therefore, we expected to obtain consistent findings across conflicting health information in the present study, regardless of whether the precise target of information differed.

Given prior research that demonstrated both perceptions of and tolerance for ambiguity were associated with pessimistic appraisals and avoidance, we hypothesized that perceived medical ambiguity and tolerance for medical ambiguity would be moderately positively correlated and that correlates of perceptions of and tolerance for medical ambiguity would be similar and in directions consistent with pessimistic appraisal and avoidance (see Table 1 for rationale and hypotheses for all included correlates).

**Table 1:** Hypotheses for the associations of ambiguity (perceived medical ambiguity and tolerance for medical ambiguity) with cancer perceptions, health‐care experiences and preferences, and information‐seeking styles and beliefs

Outcome	Rationale for inclusion	Hypotheses
Cancer perceptions		
Perceived cancer preventability	Perceived cancer preventability, cancer risk, and cancer worry are considered pessimistic appraisals that have previously been associated with perceived ambiguity.^11^, ^13^ These variables were included to replicate findings obtained with prior iterations of HINTS data.	Lower tolerance for medical ambiguity would be associated with ***greater*** perceived cancer risk and worry and ***lower*** perceived cancer preventability.
Perceived cancer risk		
Cancer worry		
Health‐care experiences and preferences		
Health self‐efficacy	Whereas pessimistic appraisals have previously referred to perceived risk and benefits as well as perceived disease preventability,^5^, ^9^, ^15^ we expanded upon this work by examining pessimistic appraisals of either one's own ability or of medical providers’ ability in the health‐care context. Specifically, Han^12^ has suggested that patient‐centred communication could help patients handle ambiguity. Less confidence or trust in experts is also one component of lower tolerance for ambiguity,^14^ which might be related to a broader lack of trust in physicians and health‐care organizations.^16^	Lower tolerance for medical ambiguity would be associated with more pessimistic appraisals in terms of ***lower*** perceived self‐efficacy for taking care of one's health, ***lower*** perceived patient‐centred communication and ***lower*** reliance and trust in doctors.
Patient‐centred communication		
Reliance on doctors		
Trust in doctors		
Shared decision making: low chance of survival	Individuals who perceived greater ambiguity have also reported preferring to avoid making decisions.^8^	Lower tolerance for medical ambiguity would be associated with ***lower*** preferences for shared decision making.
Shared decision making: moderate chance of survival		
Engagement in medical research	Clinical trials are likely to involve ambiguity given that the effectiveness of the treatment would be unknown.	Lower tolerance for ambiguity would be associated with ***lower*** likelihood of having previously engaged in clinical trials.
Information‐seeking styles and beliefs		
Cancer information avoidance	Prior research has shown that greater perceived ambiguity was associated with lower interest in learning personalized health risk information.^9^ Therefore, we predicted that lower tolerance for ambiguity would be associated with greater cancer information avoidance and more negative appraisals.	Lower tolerance for ambiguity would be associated with ***greater*** cancer information avoidance, and ***lower*** likelihood of searching for health and cancer‐related information.
Health information seeking		
Cancer information seeking		
Cancer information‐seeking self‐efficacy	Whereas pessimistic appraisals have previously referred to perceived risk and benefits as well as perceived disease preventability,^5^, ^9^, ^15^ we expanded upon this work by examining pessimistic appraisals for information seeking in health‐care contexts.	Lower tolerance for ambiguity would be associated with ***lower*** perceived self‐efficacy for cancer information seeking and ***lower*** perceived quality of the information‐seeking process.
Quality of cancer information seeking		

Note

We did not create separate hypotheses about perceived medical ambiguity; instead, we expected that all associations would be in the same direction as those hypothesized for tolerance for medical ambiguity.

## 2 METHODS

### 2.1 Data source and study population

We used data from the National Cancer Institute's publicly available Health Information National Trends Survey (HINTS 4, Cycle 4; https://hints.cancer.gov/). This HINTS cycle was approved through expedited review by the Westat IRB and considered exempt by the US National Institutes of Health Office of Human Subjects Research Protections. Kent State University's IRB deemed this project exempt. Because data used are publicly available, some variables reported here have been examined by other authors in unrelated papers (see hints.cancer.gov). This was the first HINTS cycle that assessed tolerance for medical ambiguity. Prior HINTS cycles have only assessed perceived medical ambiguity, which was also included in the present cycle. HINTS is a nationally representative, cross‐sectional survey conducted annually or biennially since 2003. US adults ≥18 years of age are surveyed regarding their use of health and cancer‐related information. HINTS uses a complex stratified sampling design to ensure nationally representative data and oversamples African‐American and Hispanic households.^18^, ^19^ HINTS 4, Cycle 4, was conducted in 2014 through mailed surveys. A total of 3677 eligible respondents completed the survey. We restricted analyses to the 3433 respondents with non‐missing data for our key variables: tolerance for medical ambiguity and perceived medical ambiguity. Additional information regarding the data source and study population, including design and sampling procedures, is available in a methodology report.^18^ This study had no external funding source.

### 2.2 Measures

HINTS items were developed by expert consensus and underwent cognitive testing. Potential medical and cancer‐specific judgement and decision‐making correlates were selected for inclusion based on prior research on perceived ambiguity and tolerance for ambiguity^13^, ^14^, ^16^, ^17^ and subsequently grouped into three categories: cancer perceptions, health‐care experiences and preferences, and information‐seeking styles and beliefs (Table 1).

#### 2.2.1 Medical ambiguity

Measures of medical ambiguity differed in their specific medical context (i.e., medical tests/treatments and cancer prevention recommendations). However, both measures referred to conflicting information.

*Tolerance for medical ambiguity* was assessed with, ‘If experts had conflicting opinions about a medical test or treatment, I would still be willing to try it’ (1 = ‘strongly agree’ to 4 = ‘strongly disagree’). This item was taken from the six‐item AA‐Med Scale.^14^ The full scale assesses individual‐level differences in cognitive, affective, and behavioural ‘manifestations’ of ambiguity. The HINTS item was taken from the behavioural subscale that includes lower uptake of medical interventions.^14^ Higher scores indicated higher aversion or lower tolerance for medical ambiguity.

*Perceived medical ambiguity* was assessed with, ‘There are so many different recommendations about preventing cancer, it is hard to know which ones to follow’ (1 = ‘strongly agree’ to 4 = ‘strongly disagree’). After reverse scoring, higher scores indicated higher levels of perceived medical ambiguity.

#### 2.2.2 Cancer perceptions

Three items assessed cancer perceptions. *Perceived cancer preventability* was assessed with, ‘There's not much you can do to lower your chances of getting cancer’ (1 = ‘strongly agree’ to 4 = ‘strongly disagree’). Higher scores indicated greater perceived cancer preventability. *Perceived cancer risk* was assessed with, ‘How likely are you to get cancer in your lifetime?’ (1 = ‘very unlikely’ to 5 = ‘very likely’). *Cancer worry* was assessed with, ‘How worried are you about getting cancer?’ (1 = ‘not at all’ to 5 = ‘extremely’).

#### 2.2.3 Health‐care experiences and preferences

*Perceived health self‐efficacy* was assessed with, ‘Overall, how confident are you about your ability to take good care of your health?’ (1 = ‘completely confident’ to 5 = ‘not confident at all’). Following reverse scoring, higher scores indicated higher self‐efficacy.

*Perceived patient‐centred communication* was assessed as the average of seven items asking respondents to indicate how often doctors, nurses or other health professionals they had seen during the past 12 months were attentive to their needs (e.g., ‘Give you the chance to ask all the health‐related questions you had’, ‘Spend enough time with you’; 1 = ‘always’ to 4 = ‘never’; α = .94). Following reverse scoring, higher scores indicated more positive perceptions of patient‐centred communication.

*Perceived reliance on doctors* was assessed with, ‘In the past 12 months, how often did you feel you could rely on your doctors, nurses, or other health care professionals to take care of your health care needs?’ (1 = ‘always’ to 4 = ‘never’). *Perceived trust in doctors* was assessed with, ‘In general, how much would you trust information about cancer from each of the following: [A Doctor]’ (1 = ‘a lot’ to 4 = ‘not at all’). Following reverse scoring, higher scores indicated higher reliance and trust. Because the residual errors were not normally distributed, which violated the assumptions of linear regression, we dichotomized response options (reliance: 0 = ‘usually,’ ‘sometimes,’ or ‘never,’ 1 = ‘always’; trust: 0 = ‘some,’ ‘a little,’ or ‘not at all,’ 1 = ‘a lot’).

*Engagement in medical research* assessed prior participation in medical research: ‘Have you ever been in a medical research study where you got one of two treatments, such as medicines or surgery procedures?’ (coded as 0 = ‘no’, 1 = ‘yes’). We interpreted this item as reflecting decision making in the context of ambiguity; ambiguity could arise from participating in a research study for which there is insufficient information regarding treatment options, such as the treatment's effectiveness.

*Shared decision making* assessed respondents’ preferences in two hypothetical scenarios: ‘Suppose you have been diagnosed with cancer with a [low/moderate] chance of survival and [limited/several] treatment options, what role would you prefer to take in deciding your cancer treatment?’ Response options ranged from 1 = ‘I prefer to make the decision with little or no input from my doctor’ to 5 = ‘I prefer to leave all decisions about my treatment to my doctor’ with a shared decision‐making option (3 = ‘I prefer that my doctor and I share responsibility for the decision together’) and intermediate responses of 2 = ‘I prefer to make the decision after seriously considering my doctor's opinion’ and 4 = ‘I prefer my doctor to make the decision after seriously considering my opinion’. Responses of 1 and 5 were recoded as 0 = ‘no’, and responses 2 through 4 were recoded as 1 = ‘yes’ to assess whether individuals preferred shared decision making.

#### 2.2.4 Information‐seeking styles and beliefs

*Cancer information avoidance* was assessed with, ‘I'd rather not know my chance of getting cancer’ (1 = ‘strongly agree’ to 4 = ‘strongly disagree’). Following reverse scoring, higher scores indicated greater cancer information avoidance.

*Health information seeking* was assessed with, ‘Have you ever looked for information about health or medical topics from any source?’ *Cancer information seeking* was assessed with, ‘Have you ever looked for information about cancer from any source?’ Response options for both items were coded as 0 = ‘no’ or 1 = ‘yes’. *Perceived cancer information‐seeking self‐efficacy* was assessed with, ‘Overall, how confident are you that you could get advice or information about cancer if you needed it?’ (1 = ‘completely confident’ to 5 = ‘not confident at all’). Following reverse scoring, higher scores indicated higher self‐efficacy.

*Perceived quality of the cancer information‐seeking process* was the average of four items prefaced by, ‘Based on the results of your search for information on cancer from all sources.’. Participants were then asked to indicate agreement with ‘It took a lot of effort to get the information you needed,’ ‘You felt frustrated during your search for the information,’ ‘You were concerned about the quality of the information,’ and ‘The information you found was hard to understand’ (1 = ‘strongly agree’ to 4 = ‘strongly disagree’; α = .85). Scores were recoded so that higher scores indicated higher perceived quality. These items were only completed by respondents who indicated having sought out cancer‐related information (n = 1454).

#### 2.2.5 Sociodemographic factors

Standard sociodemographic factors of *age*, *sex* (coded as 0 = ‘female,’ 1 = ‘male’), *race* (coded as 0 = ‘non‐white,’ 1 = ‘white’) and *education* (treated as continuous and coded as 1 = ‘less than eight years,’ 2 = ‘eight through eleven years,’ 3 = ‘twelve years or completed high school,’ 4 = ‘post‐high school training other than college,’ 5 = ‘some college,’ 6 = ‘college graduate,’ and 7 = ‘post‐graduate’) were included as covariates in all analyses consistent with prior HINTS studies on ambiguity.^11^, ^13^, ^14^ We also controlled for *health‐care coverage* which was assessed with, ‘Do you have any kind of healthcare coverage, including health insurance, prepaid plans such as HMOs, or government plans such as Medicare?’ (coded as 0 = ‘no,’ 1 = ‘yes’).

### 2.3 Overview of analyses

Hypotheses involving tolerance for medical ambiguity were pre‐registered on Open Science Framework (https://osf.io/74rab/). Analyses involving perceived medical ambiguity were conducted post‐registration; as such, we did not have a priori hypotheses regarding the strength of associations for perceived medical ambiguity versus tolerance for medical ambiguity. In addition, multiple interaction effects were hypothesized in the pre‐registration document; however, as none obtained statistical significance, we do not report them here.

Before the data were released, the government contractor that collected the data (Westat) used hot deck imputation to replace missing values for age, sex, educational attainment, race and health‐care coverage with the value reported by a similar case. The methodology report contains more information about the imputation.^18^ Unlike multiple imputation procedures, hot deck imputation does not result in multiple data sets. By using ‘donors’ with values similar to the case with missing values to impute missing values, the hot deck approach results in a distribution similar to the distribution of values observed for respondents.

The sample size differed across analyses due to missing data on individual variables. Analyses for perceived cancer risk and worry excluded individuals who indicated a diagnosis of cancer and were instructed to skip these items (*n* = 494). We conducted analyses using SAS‐callable SUDAAN to adjust for the complex sampling design and applied statistical jackknife replicate weights to render statistical estimates nationally representative.^19^ First, we examined the association between perceived medical ambiguity and tolerance for medical ambiguity. Next, we conducted bivariate analyses to examine relationships among perceptions of and tolerance for medical ambiguity with the sociodemographic factors of age, sex, race, education, and health‐care coverage (see Table S1 for Rationale and Hypotheses). We then conducted linear (for continuous outcomes) or logistic (for dichotomous outcomes) regression analyses to examine associations among perceptions of and tolerance for medical ambiguity with the health constructs previously described, controlling for age, sex, race, education, and health‐care coverage. Finally, we conducted exploratory linear and logistic analyses, controlling for the sociodemographic variables included in primary analyses, to test whether the interaction between perceived medical ambiguity and tolerance for medical ambiguity predicted any of the health constructs. Variables on scales from 1‐4 or 1‐5 were treated as continuous. In all analyses, perceptions of and tolerance for medical ambiguity were treated as independent variables.

## 3 RESULTS

Descriptive information regarding respondent characteristics is reported in Table 2. Most respondents were white (78%), about half were female, and the average age was 47 years (*SE* = 0.11). As shown in Table 2, more individuals on average would be willing to try (60.7%) versus not try (39.3%) a medical test or treatment for which experts have conflicting opinions. Despite this tolerance for medical ambiguity, most respondents agreed (74.6%) versus disagreed (25.4%) with the statement indicating perceived medical ambiguity about cancer prevention options. Importantly, tolerance for medical ambiguity (*M* = 2.44, *SE* = 0.03) and perceived medical ambiguity (*M* = 2.91, *SE* = 0.02) were not associated at the bivariate level (*r *= .01, *P *= .681, 95% Confidence Interval (CI) = −0.05, 0.08) or when controlling for sociodemographic factors (*B = *0.01*, SE = *0.03*, t*(49)=0.22, 95% CI = −0.06, 0.07, *P *= .827), suggesting that these two constructs are empirically distinguishable. All other descriptive statistics (ie mean, standard error and range) and correlations among items assessing tolerance for medical ambiguity, perceived medical ambiguity, cancer perceptions, health‐care experiences and preferences, and information‐seeking styles and beliefs are in Table S2.

**Table 2:** Distribution and weighted percentages of sociodemographic characteristics, tolerance for medical ambiguity, and perceived medical ambiguity of Health Information National Trends Survey 4, Cycle 4 respondents (n = 3433)

Sociodemographic variables	*M*	*SE*	Range
Age (y)	46.50	0.11	18‐98

	*n*	Weighted frequency	Weighted percentage
Sex			
Female	2047	115 280 306	51.00
Male	1386	110 777 280	49.00
Race			
White	2491	176 389 113	78.03
Non‐white	942	49 668 473	21.97
Education			
Less than 8 y	82	6 969 339	3.08
8 through 11 y	195	17 268 360	7.64
12 y or completed high school	637	40 841 228	18.07
Post‐high school training other than college	276	16 896 516	7.47
Some college	791	51 712 819	22.88
College graduate	885	58 278 726	25.78
Post‐graduate	567	34 090 597	15.08
Health‐care coverage			
Yes	3037	197 780 048	87.49
No	396	28 277 538	12.51
Tolerance for medical ambiguity (‘If experts had conflicting opinions about a medical test or treatment, I would still be willing to try it’)			
Strongly agree	382	22 382 913	9.90
Somewhat agree	1687	114 795 434	50.75
Somewhat disagree	810	55 445 061	24.53
Strongly disagree	554	33 434 178	14.79
Perceived medical ambiguity (‘There are so many different recommendations about preventing cancer, it is hard to know which ones to follow’)			
Strongly disagree	291	17 443 078	7.72
Somewhat disagree	614	40 041 160	17.71
Somewhat agree	1725	113 176 847	50.07
Strongly agree	803	55 396 500	24.51

Note

Tolerance for medical ambiguity: *M* = 2.44, *SE* = 0.03, range = 1‐4.

Perceived medical ambiguity: *M* = 2.91, *SE* = 0.02, range = 1‐4.

We next examined the associations of tolerance for medical ambiguity and perceived medical ambiguity with sociodemographic factors and health constructs (Tables 3 and 4).

**Table 3:** Multivariate associations of tolerance for medical ambiguity and perceived medical ambiguity with continuous measures of cancer perceptions, health‐care experiences and preferences, and information‐seeking styles and beliefs

Outcome	*n*	Tolerance for medical ambiguity^a^					Perceived medical ambiguity				
		*β*	*SE*	*t*(49)	95% CI	*P* value	*β*	*SE*	*t*(49)	95% CI	*P* value
Cancer perceptions											
Perceived cancer preventability	3412	0.04	0.03	1.40	−0.02, 0.10	.167	−0.35	0.02	−15.53	−0.40, −0.30	<.001
Perceived cancer risk	2889	−0.06	0.03	−1.92	−0.13, 0.00	.061	0.04	0.03	1.28	−0.02, 0.11	.207
Cancer worry	2925	−0.12	0.04	−2.99	−0.21, −0.04	.004	0.06	0.06	1.06	−0.05, 0.17	.295
Health‐care experiences and preferences											
Health self‐efficacy	3347	0.05	0.03	1.55	−0.01, 0.11	.127	−0.08	0.03	−3.13	−0.14, −0.03	.003
Patient‐centred communication	2794	−0.04	0.03	−1.17	−0.10, 0.03	.246	−0.04	0.02	−1.91	−0.08, 0.00	.061
Information‐seeking styles and beliefs											
Cancer Information avoidance	3399	0.04	0.04	1.08	−0.03, 0.11	.284	0.20	0.03	6.91	0.14, 0.25	<.001
Cancer information‐seeking self‐efficacy	3372	−0.05	0.03	−1.62	−0.12, 0.01	.111	−0.14	0.03	−4.09	−0.20, −0.07	<.001
Quality of cancer information seeking	1454	−0.07	0.04	−1.86	−0.14, 0.05	.069	−0.17	0.03	−5.43	−0.23, −0.11	<.001

Note

All analyses were linear regressions that controlled for sociodemographic variables of age, sex, race and health‐care coverage.

a Higher scores indicated lower tolerance for medical ambiguity or higher levels of aversion.

**Table 4:** Multivariate associations of tolerance for medical ambiguity and perceived medical ambiguity with dichotomous measures of health‐care experiences, and preferences and information‐seeking styles and beliefs

Outcome	*n*	Tolerance for medical ambiguity^a^			Perceived medical ambiguity		
		OR	95% CI	*P* value	OR	95% CI	*P* value
Health‐care experiences and preferences							
Reliance on doctors	2873	0.94	0.79, 1.11	.451	0.82	0.72, 0.94	.004
Trust in doctors	3358	0.78	0.68, 0.91	.002	0.95	0.81, 1.12	.547
Engagement in medical research	3345	0.68	0.51, 0.89	.007	1.04	0.76, 1.42	.814
Shared decision making low chance of survival	3396	0.97	0.76, 1.24	.813	0.87	0.71, 1.06	.163
Shared decision making moderate chance of survival	3377	0.92	0.65, 1.29	.616	0.97	0.73, 1.30	.846
Information‐seeking styles and beliefs							
Health information seeking	3404	0.80	0.69, 0.94	.006	0.95	0.78, 1.17	.637
Cancer information seeking	2806	0.91	0.78, 1.07	.245	0.94	0.82, 1.09	.413

Notes

All analyses were logistic regressions that controlled for sociodemographic variables of age, sex, race and health‐care coverage. Confidence intervals are calculated for the Odds Ratio. *P* values are calculated for the Wald statistic.

a Higher scores indicated lower tolerance for medical ambiguity or higher levels of aversion.

### 3.1 Tolerance for medical ambiguity

#### 3.1.1 Sociodemographic factors

Consistent with hypotheses, lower tolerance for ambiguity was reported by females (*M* = 2.51; *SE* *= *0.04), participants identifying as non‐white (*M* = 2.57; *SE* *= *0.05), and those without health‐care coverage (*M* = 2.63; *SE* *= *0.10) compared to males (*M* = 2.37; *SE* *= *0.04; *t*(49) = −2.73, *P *= .009, 95% CI = −0.25, −0.04), participants identifying as white (*M* = 2.41; *SE* *= *0.03; *t*(49) = −3.22, *P *= .002, 95% CI = −0.27, −0.06), and those with health‐care coverage (*M* = 2.42; *SE* *= *0.02; *t*(49) = −2.09, *P *= .042, 95% CI = −0.42, −0.01), respectively. Reporting a lower level of educational attainment was also associated with lower tolerance for ambiguity (*r *= −0.09, *P *< .001, 95% CI = −0.14, −0.05). Contrary to hypotheses, age was not significantly associated with tolerance for ambiguity (*r *= −.05, *P *= .085, 95% CI = −0.11, 0.01).

#### 3.1.2 Cancer perceptions

Contrary to hypotheses, participants with lower tolerance for ambiguity reported *lower* cancer worry. Tolerance for medical ambiguity was not associated with perceived cancer risk or perceived cancer preventability.

#### 3.1.3 Health‐care experiences and preferences

Consistent with hypotheses, participants with lower tolerance for ambiguity indicated lower trust in receiving information about cancer from their doctor and were less likely to have engaged in medical research. No other associations were statistically significant.

#### 3.1.4 Information‐seeking styles and beliefs

As predicted, participants with lower tolerance for ambiguity reported lower likelihood of seeking health information, but there was no association with likelihood of seeking information about cancer specifically. The associations with cancer information avoidance and perceived quality of and self‐efficacy for seeking cancer information were not statistically significant.

### 3.2 Perceived medical ambiguity

#### 3.2.1 Sociodemographic factors

As expected, participants with lower levels of educational attainment reported greater perceived medical ambiguity, *r* = −.09, *P* = .002, 95% CI = −0.15, −0.04. However, perceived medical ambiguity did not significantly differ as a function of sex (*t*(49) = 0.34, *P* = .734, 95% CI = −0.08, 0.11), race (*t*(49) = 0.82, *P* = .415, 95% CI = −0.08, 0.18), health‐care coverage (*t*(49) = −0.46, *P* = .648, 95% CI = −0.21, 0.13) or age (*r* = .00, *P* = .994, 95% CI = −0.06, 0.06).

#### 3.2.2 Cancer perceptions

Consistent with hypotheses and prior research, participants who perceived more medical ambiguity also perceived cancer as being less preventable. However, contrary to hypotheses and prior research, perceived medical ambiguity was not significantly associated with perceived cancer risk or worry.

#### 3.2.3 Health‐care experiences and preferences

As predicted, participants who perceived greater medical ambiguity indicated lower health self‐efficacy and lower beliefs that they could rely on their doctors to take care of their health‐care needs. No other associations were statistically significant.

#### 3.2.4 Information‐seeking styles and beliefs

As expected, participants who perceived more medical ambiguity reported greater desire to avoid knowing their cancer risk and lower self‐efficacy for seeking cancer information and perceived the quality of their cancer information‐seeking process as worse. However, perceived medical ambiguity was not significantly associated with likelihood of seeking health or cancer‐specific information.

#### 3.2.5 Interaction of perceived medical ambiguity and tolerance for medical ambiguity

We conducted 15 exploratory regression analyses testing whether tolerance for medical ambiguity moderated the effect of perceived medical ambiguity on health constructs. The interaction term reached statistical significance (*P *< .05) for only two variables: perceived cancer preventability and cancer worry. However, neither effect reached the adjusted statistical significance value of *P *= .003 resulting from a Bonferroni correction for the number of analyses tested (0.05/15). In addition, these effects were not predicted a priori and thus we do not describe them further.

## 4 DISCUSSION

Conflicting information and expert opinions will continue to be prevalent in medical decision‐making contexts. However, relatively little is known about the relationship between perceived medical ambiguity and having low tolerance for medical ambiguity or about medical and cancer‐specific judgement and decision‐making correlates of these constructs. Previously, researchers have inferred that ‘ambiguity aversion’ occurs when individuals who reported greater perceived ambiguity in medical contexts also reported pessimistic appraisals—for example, about their disease risk—and avoidance behaviours.^4^, ^8^, ^11^ Therefore, we hypothesized that perceived medical ambiguity and tolerance for medical ambiguity would be moderately positively associated and show similar associations with multiple related health constructs.

Contrary to these hypotheses, perceived medical ambiguity and tolerance for medical ambiguity were not associated, supporting the idea that people can be averse to ambiguity but not perceive it and that people can perceive ambiguity but not be averse to it. Put differently, perceiving and tolerating medical ambiguity appear to be conceptually and empirically distinct. This finding is consistent with prior work^9^ in which perceived ambiguity about genomic sequencing information was distinct from tolerance for medical ambiguity when the latter was assessed with the full AA‐Med scale.^14^ In the present study, items indicative of pessimistic appraisal and avoidance across three categories—cancer perceptions; health‐care experiences and preferences; and information‐seeking styles and beliefs—differed in how they were associated with perceptions of and tolerance for medical ambiguity, and no individual construct was associated with both constructs. Although we expected some separability, we were surprised that the constructs were not significantly related. As previously noted, in prior correlational data,^11^, ^13^, ^17^ ambiguity aversion is often inferred when the direction of the associations among perceived ambiguity and health cognitions and intentions is consistent with pessimistic appraisal and avoidance. For example, if perceived ambiguity and perceived cancer risk are positively correlated, researchers might infer the presence of ambiguity aversion (i.e., perceiving one's risk as higher and thus worse). Although a prior study noted a weak, albeit statistically significant (*r *= .124), correlation between perceived ambiguity about potential genomic sequencing results and ambiguity aversion, 9 the present study presents the first concerted effort to empirically disentangle the two constructs, including their association with other health constructs.

We also found that most associations between perceptions of and tolerance for medical ambiguity with perceived cancer risk, cancer worry, and perceived cancer preventability were not significant. Surprisingly, and in contrast to prior findings that greater perceived ambiguity was associated with *greater* cancer worry,^11^, ^13^ participants lower in tolerance for medical ambiguity reported significantly *lower* cancer worry. Lower levels of cancer worry might be an avoidance strategy, and thus consistent with avoidance behaviour in ambiguity aversion. Further, those lower in tolerance for ambiguity did not perceive their cancer risk as higher or cancer as less preventable. Also surprisingly, the associations of perceived ambiguity with perceived cancer risk and worry^11^, ^13^ were not replicated. However, individuals who perceived greater ambiguity reported significantly lower perceived cancer preventability, which replicated associations found using earlier HINTS cycles.^11^, ^13^

One potential explanation as to why the associations between perceived medical ambiguity and perceived cancer risk and worry did not replicate is unidentified moderators. With respect to perceived cancer risk specifically, recent data suggested that ambiguity and scientific uncertainty are sometimes associated with *lower* perceived risk.^4^, ^20^ In one study, participants who read about a hypothetical health pandemic and then were exposed to ambiguous rather than unambiguous information about a vaccine reported *lower* risk perceptions.^4^ Another study similarly found that individuals who read ambiguous rather than unambiguous health information about electronic cigarettes reported lower risk perceptions.^20^ Thus, one possible explanation for the null association between ambiguity constructs and perceived cancer risk in the present study is that there are important moderators such that for some individuals, ambiguity is associated with greater perceived risk, and for others, it is associated with lower perceived risk.^12^, ^15^ The nature of the ambiguity may also matter, such as whether it refers to ambiguity about a specific medical test or behaviour (as in some previous studies^4^, ^20^) or cancer prevention more broadly (as in HINTS). Empirical research is needed to examine individual differences or situational contexts that predict when tolerance for or exposure to ambiguous information leads to increased or decreased risk perceptions.

Our findings that participants higher in perceived medical ambiguity reported lower perceived health and information‐seeking self‐efficacy and greater cancer information avoidance are consistent with the extended parallel processing model (EPPM).^21^, ^22^, ^23^, ^24^ Ambiguous information has been conceptualized as threatening,^25^ and the EPPM posits that when confronted with threatening information, people can engage in ‘danger control’ to reduce the threat or ‘fear control’ to reduce negative emotions about the threat.^22^, ^23^, ^24^ Information *seeking* has been conceptualized as danger control because it can reduce the threat itself, whereas information *avoidance* may function as fear control by reducing negative emotions.^21^ Here, participants who perceived greater medical ambiguity reported greater cancer information avoidance—a fear control response—and lower self‐efficacy for seeking cancer information and lower quality of the cancer information‐seeking process. There was, however, no association between perceived ambiguity and seeking information about health or cancer. Additionally, prior research has shown that individuals lower in self‐efficacy are more likely to activate fear control responses, whereas those higher in self‐efficacy are more likely to activate danger control responses.^22^, ^23^, ^24^ Thus, our data suggest that people who perceive medical ambiguity may respond to this threat by attempting to reduce negative emotion rather than proactively attempting to reduce risk.

We also found that individuals lower in tolerance for medical ambiguity were less likely to have participated in medical research and to have searched for health information. Although less participation in medical research is not necessarily detrimental, it is important to consider whether people who are less tolerant of medical ambiguity might be less likely to participate in other potentially ambiguous medical situations. Considering that medical recommendations are often ambiguous, people with lower tolerance for ambiguity might respond to this information by avoiding medical procedures. This is especially concerning because both measures of medical ambiguity were significantly associated with either lower trust in doctors or lower reliance on doctors, consistent with findings that ambiguous information about a vaccine during a hypothetical health pandemic decreased trust in health officials.^4^

### 4.1 Limitations

There were several limitations of the present study. First, cross‐sectional data preclude causal inferences. Second, many constructs—including perceived medical ambiguity and tolerance for medical ambiguity—were assessed with single items. Although single items and multi‐item scales can have comparable psychometric properties,^26^, ^27^ single items can be less reliable, potentially leading to weakened effects or the null associations observed here.^28^ However, this does not explain why we did not replicate prior associations shown using the identical single‐item measure of perceived ambiguity in previous HINTS cycles.^11^, ^13^ Third, findings may be attributed to conceptual differences among the constructs themselves. Whereas perceived medical ambiguity has been measured in prior studies with this same item we used,^11^, ^13^ this item has also been labelled as assessing fatalism^29^ and information overload.^30^ Despite multiple conceptual interpretations, we are confident this item assesses perceived ambiguity because it assesses awareness of conflicting information, a common source of ambiguity.

Further, there were conceptual differences between the specificity of the medical domain referenced in the items assessing tolerance for ambiguity and perceived ambiguity. The item assessing tolerance for ambiguity referred to conflicting opinions regarding medical tests and treatments, whereas the item assessing perceived ambiguity referred to conflicting cancer prevention recommendations. Despite these conceptual differences between the precise medical context, both measures of ambiguity referred to conflicting medical information. In the present study, we concluded that perceived medical ambiguity and tolerance for medical ambiguity were distinct because the associations of both measures differed across the general medical and cancer‐specific judgement and decision‐making correlates that were assessed. In particular, the measure of ambiguity that was specific to a cancer context (i.e., perceived medical ambiguity) was associated with two general medical judgement and decision‐making correlates, whereas the measure of ambiguity that was specific to medical tests and treatments (i.e., tolerance for medical ambiguity) was associated with two cancer‐specific judgement and decision‐making correlates.

However, the different pattern of effects across the two ambiguity constructs could have resulted from one item referring to ambiguity about cancer prevention and the other to medical tests and treatments. More generally, within a person, level of tolerance for ambiguity could differ based on severity of the target. For example, a person might have more tolerance for ambiguity about cold prevention recommendations—a disease typically low in severity—than for ambiguous cancer prevention recommendations—a disease higher in severity. Research is needed to test the distinctiveness of perceived ambiguity and tolerance for ambiguity when both are assessed with reference to the same target (e.g., cancer prevention or medical tests and treatments) and to determine whether effects generalize to other medical domains differing in severity. Future factor analytic work using multi‐item assessments of perceptions of and tolerance for medical ambiguity will aid in determining the distinctiveness of these constructs.

## 5 CONCLUSION

The present study distinguished between perceived medical ambiguity—assessed about cancer prevention recommendations—and tolerance for medical ambiguity—assessed about medical tests and treatments—and suggests that these constructs are distinct. Across categories, lower tolerance for medical ambiguity was associated with lower cancer worry, less trust in receiving information about cancer from doctors, less engagement in medical research, and lower likelihood of searching for health information. Greater perceived medical ambiguity was associated with lower perceived cancer preventability, lower health self‐efficacy, lower reliance on doctors, greater cancer information avoidance, lower information‐seeking self‐efficacy, and lower perceived quality of cancer information seeking. Thus, on the whole, greater perceptions and lower tolerance for medical ambiguity were indeed associated with more pessimistic appraisals and greater avoidance. Additional studies might examine whether these constructs are distinct when measured within the same precise medical context and using multiple‐item measures. Future research should also continue to elucidate the nature of perceptions of medical ambiguity and aversive responses to such medical ambiguity reflecting individual‐level differences in tolerance, and how these two constructs might differ from each other and manifest across important health constructs.

## CONFLICT OF INTEREST

The authors declare that there is no conflict of interest.

## Data Availability

We used data from the National Cancer Institute's Health Information National Trends Survey (HINTS 4, Cycle 4), which are publicly available and can be accessed at https://hints.cancer.gov/.
