# Supplementary material for: Evidence that perceptions of and tolerance for medical ambiguity are distinct constructs: An analysis of nationally representative US data
Source: Health Expect. 2020 Feb 25;23(3):603–13. doi: 10.1111/hex.13037 (PMC7321721; doi:10.1111/hex.13037)
Supplement: Supplementary file 1 [file HEX-23-603-s001.docx]

Supplemental Table 1. Hypotheses for measures of ambiguity (perceived medical ambiguity and tolerance for medical ambiguity) with sociodemographic factors

| **Outcome** | **Rationale for Inclusion** | **Hypotheses** |
| --- | --- | --- |
| **Sociodemographic Factors** |  |  |
| Age | We expected associations between tolerance for ambiguity with age, education, gender, and race to be consistent with prior research on both tolerance for medical ambiguity and perceived medical ambiguity.^14,17,32^ | We hypothesized that participants who reported lower tolerance for medical ambiguity would be older, report lower educational attainment, and be more likely to be female and non-White. |
| Education |  |  |
| Gender |  |  |
| Race |  |  |
| Healthcare Coverage | We included healthcare coverage as an exploratory analysis and did not have hypotheses regarding whether tolerance for medical ambiguity would be associated with healthcare coverage. | |

*Note.* We did not create separate hypotheses about perceived medical ambiguity; instead, we expected that all hypotheses would be the same as our hypotheses using the measure of tolerance for medical ambiguity.

Supplemental Table 2. Descriptives of and correlations among measures of medical ambiguity and cancer perceptions, healthcare experiences and preferences, and information seeking styles and beliefs

|  | 1 | 2 | 3 | 4 | 5 | 6 | 7 | 8 | 9 | 10 | 11 | 12 | 13 | 14 | 15 | 16 | 17 |
| --- | --- | --- | --- | --- | --- | --- | --- | --- | --- | --- | --- | --- | --- | --- | --- | --- | --- |
| 1. Tolerance for Medical Ambiguity^1^ | - | .01 | .01 | -.07** | -.07* | .04 | -.05 | -.03 | -.09** | -.07** | -.02 | -.03 | .05 | -.09** | -.05 | -.06* | -.07^ |
| 2. Perceived Medical Ambiguity |  | - | -.34** | .05^ | .06 | -.10** | -.05^ | -.08** | -.02 | .01 | -.05* | -.02 | .18** | -.03 | -.04 | -.12** | -.21** |
| 3. Perceived Cancer Preventability |  |  | - | -.02 | -.07* | .11** | .05^ | .05 | .09** | -.04 | .08** | .07* | -.24** | .15** | .04 | .14** | .17** |
| 4. Perceived Cancer Risk |  |  |  | - | .28** | -.08* | -.03 | -.02 | .05^ | .02 | .08^ | .08** | -.08** | .15** | .15** | .06^ | .01 |
| 5. Cancer Worry |  |  |  |  | - | -.14** | -.08* | -.07^ | -.01 | .04 | .04 | .02 | -.13** | .08* | .16** | -.06^ | -.07 |
| 6. Health Self-Efficacy |  |  |  |  |  | - | .24** | .22** | .13** | -.04* | -.01 | .01 | -.06^ | -.00 | .05 | .27** | 0.21** |
| 7. Patient Centered Communication |  |  |  |  |  |  | - | .54** | .26** | .03 | .01 | -.00 | -.03 | -.05^ | -.00 | .19** | .29** |
| 8. Reliance on Doctors |  |  |  |  |  |  |  | - | .25** | .05* | .01 | -.01 | .03 | -.02 | -.05 | .20** | .29** |
| 9. Trust in Doctors |  |  |  |  |  |  |  |  | - | -.03 | .07* | .05* | -.07* | .02 | .02 | .27** | .17** |
| 10. Engagement in Medical  Research |  |  |  |  |  |  |  |  |  | - | -.04 | -.06^ | -.00 | .03 | .07** | .01 | -.02 |
| 11. Shared Decision Making-  Low Chance of Survival |  |  |  |  |  |  |  |  |  |  | - | .52** | -.08** | .07* | .01 | .05^ | .04 |
| 12. Shared Decision Making-  Moderate Chance of Survival |  |  |  |  |  |  |  |  |  |  |  | - | -.09** | .08* | .02 | .06* | .11* |
| 13. Information Avoidance |  |  |  |  |  |  |  |  |  |  |  |  | - | -.12** | -.11** | -.10** | -.09* |
| 14. Health Information-Seeking |  |  |  |  |  |  |  |  |  |  |  |  |  | - | - | .07^ | - |
| 15. Cancer Information-Seeking |  |  |  |  |  |  |  |  |  |  |  |  |  |  | - | -.01 | - |
| 16. Cancer Information Seeking Self Efficacy |  |  |  |  |  |  |  |  |  |  |  |  |  |  |  | - | .50** |
| 17. Quality of Cancer Information-  Seeking |  |  |  |  |  |  |  |  |  |  |  |  |  |  |  |  | - |
| *M* | 2.44 | 2.91 | 2.99 | 3.14 | 2.55 | 3.85 | 3.36 | 0.50 | 0.73 | 0.05 | 0.91 | 0.95 | 2.00 | 0.82 | 0.54 | 3.83 | 2.87 |
| *SE* | 0.03 | 0.02 | 0.02 | 0.03 | 0.03 | 0.02 | 0.02 | 0.02 | 0.01 | 0.01 | 0.01 | 0.01 | 0.03 | 0.01 | 0.02 | 0.03 | 0.03 |
| Range | 1-4 | 1-4 | 1-4 | 1-5 | 1-5 | 1-5 | 1-4 | 0-1 | 0-1 | 0-1 | 0-1 | 0-1 | 1-4 | 0-1 | 0-1 | 1-5 | 1-4 |

*Notes.* ^*p*≤.10, **p*≤.05, ***p*≤.01

Correlations are not provided among variables of health information-seeking, cancer information-seeking, and quality of the cancer information-seeking process because participants were not asked about cancer information-seeking if they did not report engagement in health information-seeking and participants were not asked about the quality of the cancer information-seeking process if they did not report engagement in cancer information-seeking.

^1^Higher scores indicated lower tolerance for medical ambiguity, or higher levels of aversion.
